# Supplementary material for: Prevalence and predictors of Aspergillus seropositivity and chronic pulmonary aspergillosis in an urban tertiary hospital in Sierra Leone: A cross-sectional study
Source: PLoS Negl Trop Dis. 2023 Jul 17;17(7):e0011284. doi: 10.1371/journal.pntd.0011284 (PMC10374077; doi:10.1371/journal.pntd.0011284)
Supplement: Attachment — Submitted filename: PLOS NTD RESPONSE TO REVIEWERS 10.05.23.docx [file pntd.0011284.s002.docx]

**Reviewer #1**

1. Comment: In this work, Lakoh and colleagues have undertaken a study to assess the predictors of chronic pulmonary aspergillosis in Sierra Leone. The author's have tested Aspergillus seropositivity and CPA diagnosis defined by the GAFFI panel. The authors then used the demographic, clinical and radiographic variables for their utility as CPA predictors. The authors have identified current or prior TB as an independent predictor for CPA for their patient cohort in Sierra Leone.

*Thank you.*

2. Comment: Overall, the employed methods are sound, however, this reviewer wondered why other underlying conditions such as ABPA, asthma, pneumonia were also not considered in the authors' analyses. As it has been documented previously that the aforementioned conditions can be predisposing to CPA, can these conditions can also serve as predictors?

*We included asthma, COPD and pneumonia (or bronchitis) (Table 3). Patients with asthma had 7.99 higher odds of being colonized by Aspergillus spp. (Table 3). There is a strong association between asthma and Aspergillus colonization. There was no association between Aspergillus colonization and COPD or pneumonia (Table 4). Adding the variables such as asthma, COPD and pneumonia/bronchitis did not change the predictors for CPA.We have added this information in the manuscript.*

3. The authors have described the results succinctly, however, the tables are organized in a confusing way; for examples, if footnotes are provided regarding how the data are organized, that would help the readers. Furthermore, Table 3 requires corrections; it is missing values.

*We have split off the radiological findings into a new table 3, to reduce the length of Table 2. The ‘missing values’ in Tables 3 and 4 (now tables 4 and 5) are appropriate in the multivariate analysis columns.*

4. Overall, this study aims to characterize CPA prevalence and its predictors in Sierra Leone. In the light of increasing awareness towards fungal diseases, this study serves a critical unmet need; however, considering the previously established links between TB and CPA by multiple groups, the lack of novelty decreases this reviewer's enthusiasm.

*We agree that there is previous evidence suggesting a link between TB and CPA. However, there is little or no information on CPA in Sierra Leone and even in neighbouring countries like Liberia and Guinea at the moment. Thus, we have highlighted the need to train healthcare workers and strengthen the diagnosis, management and prevention of CPA in Sierra Leone and similar countries.*

Reviewer #2

1. There are no concerns about the ethical or regulatory requirements being met and statistical analyses are correctly applied. The sample size collected was actually larger than required based on power analysis and the population of patients recruited is clearly described.

*Thank you*

2. The analyses exactly match the proposed plan and all results are clearly described. The figure and tables are high quality and arrange the data logically.

*Thank you*

3. The conclusions are clearly supported by the data presented and limitations and alternative interpretations are provided. The authors provide an excellent Discussion section addressing not only the interpretations of the data but also how it advances our understanding and what aspect will need to be further studied.

*Thank you*
